# Validation of a specific measure to assess health-related quality of life in patients with schizophrenia and bipolar disorder: the 'Tolerability and quality of life' (TOOL) questionnaire

**DOI:** 10.1186/1744-859X-10-6

**Published:** 2011-03-11

**Authors:** Angel L Montejo, Javier Correas Lauffer, Jesús Cuervo, Pablo Rebollo, Luis Cordero, Teresa Diez, Jorge Maurino

**Affiliations:** 1 Department of Psychiatry, Hospital Universitario de Salamanca, Salamanca, Spain; 2 Department of Psychiatry, Hospital del Henares, Coslada, Madrid, Spain; 3 BAP Health Outcomes Research, Oviedo, Spain; 4 Value Demonstration Unit, AstraZeneca Medical Department, Madrid, Spain; 5 Neuroscience Area, AstraZeneca Medical Department, Madrid, Spain

## Abstract

**Background:**

Perception of quality of life may differ depending on the perspective. The aim of the study was to assess the psychometric properties of the Spanish version of the 'TOlerability and quality Of Life' (TOOL) questionnaire, a specific self-rated instrument to evaluate the impact of side effects of antipsychotic drugs on health-related quality of life (HRQoL). The questionnaire consists of eight items answered on a four-point Likert scale.

**Methods:**

A psychometric study was conducted with clinically stable outpatients with schizophrenia and bipolar disorder under antipsychotic treatment. The translation and cultural adaptation of the questionnaire was performed according to international standards. Internal consistency using the Cronbach α coefficient and test-retest reliability using the intraclass correlation coefficient (ICC) was used to assess the reliability of the instrument. Patients completed generic and specific measures of quality of life and clinical severity.

**Results:**

A total of 238 patients were analysed, with a mean age of 42 years (SD 10.9). The mean completion time was 4.9 min (SD 4.4). Internal consistency and intraclass correlation coefficient were adequate (Cronbach α = 0.757 and ICC = 0.90). Factorial analysis showed a unidimensional structure (a single eigenvalue >1, accounting for 39.1% of variance). Significant Spearman's rank correlations between the TOOL and both generic and specific measures were found. The questionnaire was able to discriminate among the Clinical Global Impression - Severity scores (Mann-Whitney U test, *P *< 0.001).

**Conclusions:**

The TOOL questionnaire shows appropriate feasibility, reliability, and discriminative performance as a patient-reported outcome. TOOL constitutes a valuable addition to measure the impact of adverse events of antipsychotic drugs from the patient perspective.

## Background

Schizophrenia and bipolar disorder are worldwide prevalent and severe mental diseases [1,2]. Newer antipsychotic treatments have proven useful in reducing both relapses and the severity of symptoms [3]. However, weight gain, extrapyramidal symptoms, sexual dysfunction, or sedation are quite common side effects among patients under antipsychotic treatment [4]. The occurrence of these symptoms may affect patient adherence to medication, leading to discontinuation, limiting treatment effectiveness, and increasing both personal and social costs [5,6]. Therefore, when comparing alternative therapies, side effects and their impact on patient health-related quality of life (HRQoL) could be of great importance in order to define the most efficient antipsychotic drug treatment [3,7].

The systematic assessment of patient perspective may provide valuable information that could be lost when relying only on clinical evaluation. In chronic conditions such as schizophrenia and bipolar disorder, there are advantages in using patient-reported measures to understand complex needs and improve alliances between patients and clinicians [8]. The importance of involving patients in their own healthcare, and of patient-reported assessments, is increasingly recognised [9].

Many efforts have been made to develop or validate specific instruments to assess patient affectation within different domains [10-13]. Regarding side effects, the most widely used and specific measure is the Udvalg for Kliniske Undersøgelser (UKU) side effects scale [14]. This questionnaire, filled out by clinicians or patients, comprises 56 items that refer to psychic, neurological, autonomical and others effects. Despite this scale, more specific tools in terms of HRQoL are still needed to afford greater insight in describing and grading the impact of side effects associated to antipsychotic drugs [15,16]. A brief instrument is preferred for use in clinical practice and in investigations such as clinical management studies or clinical trials comparing the effectiveness of treatments.

A specific measure to assess HRQoL impairment related to adverse events of antipsychotic drugs has been previously developed and validated in Sweden: the 'TOlerability and quality Of Life' (TOOL) questionnaire [17]. This self-rated measure reflects the subjective perception of side effects in patients treated with antipsychotic medication. It comprises eight attributes and has four levels per domain (Likert scale: 1 = minimum to 4 = maximum impact). These domains are mood (worry-upset), function capabilities, and several adverse events frequently associated with antipsychotic treatment (fatigue-weakness, weight gain, stiffness-tremor, physical restlessness, sexual dysfunction, and dizziness-nausea). In contrast to the Drug Attitude Inventory [10] or the Subjective Well-being under Neuroleptic scale [11], the TOOL questionnaire was specifically designed to identify from the patient perspective the most common adverse events of both typical and atypical antipsychotic drugs [4,18].

The aim of the present study was to evaluate the linguistic adaptation and psychometric validation into Spanish of the TOOL questionnaire for the assessment of side effects in patients treated with antipsychotic medication and their impact on health-related quality of life.

## Methods

### Linguistic adaptation of the TOOL questionnaire

Forward/backward translations of the original TOOL questionnaire were completed by expert translators. Firstly, three independent Spanish experts translated the original version in Swedish into Spanish. Experts examined and compared these three different versions in order to reach a single one by consensus (intermediate version 1.0). Secondly, one Swedish expert translated this intermediate Spanish version again into Swedish (backward translation) and compared his results with the original version to ensure conceptual equivalence (intermediate version 2.0). Finally, all the expert translators participated in the proof reading test of the intermediate version 2.0, and the final Spanish version was thus established.

Subsequently, the Spanish version was reviewed by a panel of experts (five psychiatrists and one general practitioner specialised in HRQoL). According to expert criterion, three items were modified to facilitate patient comprehension: mood, physical restlessness, and dizziness-nausea. Next, 40 clinically stable patients (20 with schizophrenia and 20 with bipolar disorder) filled out the Spanish version of the TOOL. They were also asked to review this version in terms of comprehension (C) and importance (I) using a scale ranged from 0 (lowest level of C/I) to 4 (highest level of C/I). All the items scored higher than two points (threshold) in both scales. Consequently, the Spanish version of the TOOL questionnaire was ready for validation (Additional file 1).

### Psychometric validation of the TOOL questionnaire

A multicentre, non-interventional psychometric study was conducted. The study was carried out at 60 psychiatric centres throughout Spain, and was approved by the institutional review board of the University Hospital of Salamanca (NCT00692133).

Participants were outpatients treated in community healthcare centres. Eligibility criteria included being at least 18 years old, having a Diagnostic and Statistical Manual of Mental Disorders, 4th Edition, Text Revision (DSM-IV-TR) diagnosis of schizophrenia or bipolar disorder as established by the Structured Clinical Interview for DSM-IV, being clinically stable (defined as having had no changes in severity or new treatments initiated in the last month), and taking a single oral antipsychotic medication. After complete description of the study to the participants, written informed consent was obtained.

Investigators completed the following scales: Positive and Negative Syndrome Scale (PANSS) [19] (only to patients with schizophrenia), Young Mania Rating Scale (YMRS) [20] (only to patients with bipolar disorder), Montgomery-Asberg Depression Scale (MADRS) [21], Clinical Global Impression - Severity scale (CGI-S) [22], and UKU Side Effect Rating scale (UKU) [14].

**Figure 1:**
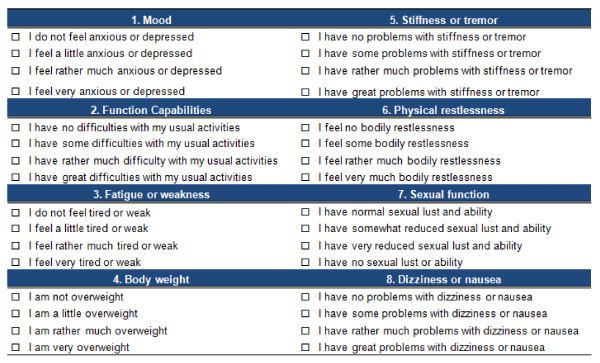
**The 'TOlerability and quality Of Life' (TOOL) questionnaire**.

Patients completed the following instruments: (1) The EuroQol 5-Dimensions (EQ-5D) [23] and Short Form 6-Dimensions (SF-6D) [24]: taking into account that the aim of this work is not to obtain utilities but to measure patient HRQoL, in these multidimensional scales we used the unweighted scores. In both cases, the higher the scores, the better the HRQoL, and *vice versa*. (2) The Spanish version of the TOOL questionnaire. The full items of the TOOL questionnaire are shown in Figure 1.

Retesting was carried out 2 to 3 weeks after the first visit. In order to analyse the psychometric properties of the TOOL questionnaire, feasibility, reliability and validity were studied.

### Feasibility

The item response rate of the TOOL questionnaire was described and completion time was registered as well. Finally, both floor-ceiling effects were evaluated in an exploratory analysis of the answers given to the TOOL distribution and by using percentiles 25, 50 and 75 (Tukey's Hinges).

### Validity

Regarding construct validity, item-total correlation (ITC) was checked following the criterion of removing items with a score lower than 0.20 in the discrimination rate. To assess the dimensionality of the questionnaire, an exploratory factorial analysis (extraction criterion of eigenvalue >1) was also conducted. The Spearman rank correlation coefficient (r_s_) between the total score of the TOOL and those of the EQ-5 D (unweighted) and the SF-6 D (unweighted) was used to test convergent validity. Correlation was considered to be high for r_s _≥ 0.5, moderate for r_s _values between 0.3 to 0.5, and low for r_s _< 0.3. This statistic was also applied to determine the relationship between the TOOL and the MADRS, YMRS, and UKU scales. These analyses were performed differentially according to the two groups resulting from clinical diagnosis (patients with schizophrenia or bipolar disorder). In addition, r_s _was used to study the association between each of the TOOL items and SF-6 D and EQ-5 D measurements.

In order to evaluate criterion validity, patients were categorised taking into account the CGI-S to compare TOOL scores. Accordingly, the collected sample was divided into two groups: patients with no to mild involvement versus patients with moderate to severe involvement. It was hypothesised that patients with lesser involvement would obtain lower scores in the TOOL (that is, lesser side effects and a better HRQoL) than those patients with worse health status according to expert criterion. Again, a non-parametric statistic (Mann-Whitney U test) was used in these comparisons considering the skewed distribution of data.

### Reliability

The internal consistency of the questionnaire was analysed using Cronbach's α. Test-retest reliability of the scores was also examined by computing the intraclass correlation coefficient (ICC).

### Statistical analysis

The sample size was calculated taking into account a specific objective of calculating multiattribute utility function reflecting patient experience of side effects of antipsychotic therapy [25]. Thus, the determination was guided by the estimation of the utility function according to which a large number of patients is needed to maximise the precision and reduce the risk of measurement error. To this end, in the present study a sample size of about 250 patients was considered. Consequently, the number of patients required for the validation process (5 to 15 patients per item) was ensured, and hypothetical comparisons between the 2 independent groups could be established (that is, schizophrenic and bipolar patients) with 80% power and a 5% significance level (2-sided tests), involving 68 patients per group.

Data tabulation, database validation and the statistical analyses were carried out using the statistical packages SPSS (version 14.0; SPSS, Chicago, IL, USA) and Stata (version 10.0; Stata, College Station, TX, USA). For all statistical tests, a level of 0.05 was considered significant.

## Registration

This trial was registered under no. NCT00692133.

## Results

A total of 242 patients were included in the study. For the validation analysis, 238 (121 with a diagnosis of schizophrenia and 117 with bipolar disorder) were studied. The mean age of the sample was 41.9 years (SD 10.9), and 63% were males (n = 151). Sociodemographic and clinical characteristics of the sample are shown in Tables 1 and 2.

**Table 1:** Sociodemographic and clinical characteristics of the sample

Variables	Total n = 238	n (%)
Gender, male		151 (63.4)

Age, mean (SD)	41.9 (10.9)	

Median time of treatment, months (SD)	33.50 (34.88)	

Marital status, n (%):		

Single		133 (55.9)

Married		69 (29.0)

Separated		17 (7.1)

Divorced		12 (5.0)

Widowed		7 (2.9)

Living status:		

Alone		28 (11.9)

With parents		109 (46.4)

In a couple		74 (31.5)

Others		24 (10.2)

Working status:		

Working full time		44 (18.5)

Working part time		28 (11.8)

Student		5 (2.1)

Unemployed		45 (18.9)

Retired (result of disease)		89 (37.4)

Retired (age)		10 (4.2)

Others		17 (7.1)

Clinical Global Impression Severity (median score = 3.00):		

Normal		23 (9.7)

Borderline mentally ill		18 (7.6)

Mildly ill		79 (33.2)

Moderately ill		81 (34.0)

Markedly ill		32 (13.4)

Severely ill		5 (2.1)

Treatment with atypical antipsychotic drugs		227 (95.4)

**Table 2:** Clinical characteristics of the sample

						Percentiles		
						
Measures	N	Mean	SD	Minimum	Maximum	25	50	75
PANSS-positive	134	13.63	6.237	7.00	36.00	9.00	12.00	17.00

PANSS-negative	134	19.91	7.938	7.00	41.00	14.75	19.00	26.00

PANSS-combined	133	-6.11	7.569	-29.00	10.00	-11.00	-6.00	-0.50

PANSS-general psychopathology	134	32.76	12.086	16.00	78.00	22.75	31.50	40.00

YMRS	118	5.61	6.777	0.00	32.00	1.00	3.00	8.25

MADRS	231	11.30	8.026	0.00	42.00	5.00	10.00	17.00

UKU-psychic	229	1.66	1.518	0.00	7.00	0.00	1.00	3.00

UKU-autonomous	224	1.87	1.974	0.00	10.00	0.00	1.00	3.00

UKU-extrapyramidal	233	1.29	1.978	0.00	10.00	0.00	1.00	2.00

UKU-total score	173	6.83	6.019	0.00	37.00	2.50	6.00	9.00

UKU-sexual	198	2.20	2.536	0.00	13.00	0.00	1.00	4.00

TOOL	233	13.462	3.430	8.00	32.00	11.00	13.00	15.00

EQ-5 D (unweighted)	234	0.743	0.248	-0.11	1.00	0.682	0.841	1.00

SF-6 D (unweighted)	236	0.785	0.125	0.39	1.00	0.708	0.793	0.893

EQ-5 D = EuroQol 5-Dimensions; MADRS = Montgomery-Asberg Depression Scale; PANSS = Positive and Negative Syndrome Scale; SF-6 D = Short Form 6-Dimensions; TOOL = 'TOlerability and quality Of Life'; UKU = Udvalg for Kliniske Undersøgelser; YMRS = Young Mania Rating Scale.

The percentage of missing values in each of the items contained in the questionnaire was less than 5%. The mean completion time was 4.91 min (SD 4.48). A ceiling effect was found for the following items: stiffness-tremor and dizziness-nausea (more than 50% of all patients reported not being bothered at all by any of these symptoms).

ITC scores were above 0.2 in all items, and body weight was the only item with a discrimination index below 0.3: ITC worry-upset = 0.581; function capabilities = 0.598; fatigue-weakness = 0.633; weight = 0.284; stiffness-tremor = 0.401; physical restlessness = 0.505; sexual dysfunction = 0.360; dizziness-nausea = 0.345. Exploratory factorial analysis reflected a unidimensional structure of the TOOL questionnaire (eigenvalue = 3.331) (Table 3). This dimension explained 39.1% of the variance. The component matrix is shown in Table 2. Furthermore, the reliability of this structure in terms of internal consistency was found to be adequate (Cronbach α = 0.757). When test-retest reliability was examined, the results showed fairly appropriate stability in the evaluations of patient involvement when no changes in clinical status were detected by the clinicians (ICC = 0.90). Since unidimensionality had been proven, it was possible to calculate a global score summarising the patient level in each of the eight domains. A range of scores between 8 (minimum impact) to 32 (maximum impact) was obtained. This total score was also analysed with respect to generic and specific measures to test convergent and criterion validity.

**Table 3:** Spanish 'TOlerability and quality Of Life' (TOOL) questionnaire construct validity: principal component analysis and component matrix

Component	Initial eigenvalues			Extraction sums of squared loadings		
	
	Total	Percentage of variance	Cumulative percentage	Total	Percentage of variance	Cumulative percentage
1	3.131	39.140	39.140	3.131	39.140	39.140

2	0.955	11.934	51.074			

3	0.905	11.315	62.388			

4	0.846	10.570	72.959			

5	0.742	9.279	82.238			

6	0.563	7.032	89.269			

7	0.443	5.534	94.803			

8	0.416	5.197	100.000			

Component matrix: Spanish TOOL domains						

	Component 1					

Worry-upset	0.747					

Function capabilities	0.759					

Fatigue-weakness	0.789					

Weight gain	0.392					

Stiffness-tremor	0.548					

Physical restlessness	0.670					

Sexual dysfunction	0.491					

Dizziness-nausea	0.481					

Correlations between the TOOL and generic measures of HRQoL were highly-moderately negative and significant (Table 4). These results were also observed when the TOOL scores of bipolar and schizophrenic patients were compared separately (Table 4). Negative values in r_s _responded to the inverse scoring of the measures. In addition, the associations between the TOOL items and the generic measures of HRQoL (Table 4) were negative, significant and mild to high. With respect to specific measures, the TOOL questionnaire correlated moderately-highly and significantly with the MADRS. Moreover, regarding CGI-S, correlation was not only moderate and significant but also higher than the one found between these scales and the HRQoL generic measures (Table 5). Correlations between specific domains of TOOL and related UKU subscales were positive, significant and mild or moderate (Table 6).

**Table 4:** Spearman rank correlations (r_s_) between Spanish 'TOlerability and quality Of Life' (TOOL) and generic measures of health-related quality of life (HRQoL)

r_s_	Function capabilities	Fatigue-weakness	Weight gain	Stiffness-tremor	Physical restlessness	Sexual dysfunction	Dizziness-nausea	EQ-5 D (unweighted)	SF-6 D (unweighted)
Worry-upset	0.501**	0.495**	0.204**	0.230**	0.416**	0.225**	0.121	-0.532**	-0.537**

Function capabilities		0.471**	0.211**	0.207**	0.375**	0.306**	0.156*	-0.591**	-0.642**

Fatigue-weakness			0.222**	0.253**	0.409**	0.291**	0.279**	-0.557**	-0.565**

Weight gain				0.213**	0.080	0.191**	0.082	-0.225**	-0.238**

Stiffness-tremor					0.243**	0.111	0.187**	-0.328**	-0.277**

Physical restlessness						0.264**	0.090	-0.422**	-0.334**

Sexual dysfunction							0.164**	-0.406**	-0.382**

Dizziness-nausea								-0.224**	-0.158*

Spanish TOOL	-	-	-	-	-	-	-	-0.720**	-0.678**

Spanish TOOL (schizophrenia)	-	-	-	-	-	-	-	-0.716**	-0.645**

Spanish TOOL (bipolar disorder)	-	-	-	-	-	-	-	-0.720**	-0.724**

*Correlation is significant at the 0.05 level (two tailed); **correlation is significant at the 0.01 level (two tailed).

**Table 5:** Spearman rank correlations (r_s_) between generic and specific measures of health-related quality of life (HRQoL)

r_s_	EQ-5 D (unweighted)	SF-6 D (unweighted)	CGI-S	PANSS: general psychopathology	MADRS	YMRS
Spanish TOOL	-0.720**	-0.678**	0.399**	0.443**	0.578**	0.239**

EQ-5 D (unweighted)		0.722**	-0.306**	-0.362**	-0.483**	-0.209*

SF-6 D (unweighted)			-0.386**	-0.516**	-0.663**	-0.100

CGI-S				0.578**	0.601**	0.351**

PANSS: general psychopathology					0.758**	0.462

MADRS						0.321**

*Correlation is significant at the 0.05 level (two tailed); **correlation is significant at the 0.01 level (two tailed).

CGI-S = Clinical Global Impression - Severity; EQ-5 D = EuroQol 5-Dimensions; MADRS = Montgomery-Asberg Depression Scale; PANSS = Positive and Negative Syndrome Scale; SF-6 D = Short Form 6-Dimensions; TOOL = 'TOlerability and quality Of Life'; YMRS = Young Mania Rating Scale.

**Table 6:** Spearman rank correlations (r_s_) between Spanish 'TOlerability and quality Of Life' (TOOL) items and UKU subscales.

r_s_	UKU-psychic	UKU-autonomous	UKU-extrapyramidal	UKU-sexual
Worry-upset	0.372**	0.156*	0.097	0.122

Function capabilities	0.359**	0.143*	0.218**	0.217**

Fatigue-weakness	0.403**	0.220**	0.171**	0.193**

Weight gain	0.300**	0.240**	0.239**	0.147*

Stiffness-tremor	0.192**	0.160*	0.560**	0.196**

Physical restlessness	0.378**	0.202**	0.154*	0.178*

Sexual dysfunction	0.201**	0.302**	0.167*	0.605**

Dizziness-nausea	0.153*	0.206**	0.184**	0.204**

*Correlation is significant at the 0.05 level (two tailed); **correlation is significant at the 0.01 level (two tailed).

UKU = Udvalg for Kliniske Undersøgelser.

Finally, with the aim of testing criterion validity and discriminative validity of the TOOL, patients were classified depending on their CGI-S scores, yielding two groups: patients with no or only mild involvement, and patients with moderate or severe involvement. Differences between these groups of CGI-S in the TOOL and generic HRQoL scores (Mann-Whitney U test) were significant in all cases (*P *< 0.001). The results highlighted that patients with no or only mild involvement had lower TOOL scores and higher EQ-5 D and SF-6 D scores, indicating a better HRQoL (mild CGI-S: TOOL = 12.168; EQ-5 D = 0.821; SF-6 D = 0.832). In contrast, those patients with moderate-severe involvement showed higher TOOL scores and lower EQ-5 D and SF-6 D outcomes (moderate-severe CGI-S: TOOL = 14.825; EQ-5 D = 0.663; SF-6 D = 0.739).

## Discussion

Agents involved in health technology research have incorporated other important aspects to the basic aims of granting new treatments efficacy and safety, such as those related to patient subjective perception [9,26]. There is published evidence on the appropriateness and accuracy of self-assessments or self-report evaluations in patients suffering from mental chronic illness [27-29]. Despite this, however, recently published studies have focused on the side effects of antipsychotic drugs and their relationship with HRQoL by following mainly clinician criterion [30]. Few studies have tried to develop specific instruments to comprehensively quantify the impact of side effects on HRQoL based on the patient perspective. This objective should be of primary importance, taking into account the high prevalence of side effects in patients undergoing antipsychotic treatments and their relationship with adherence to treatment [5].

Our results show that the TOOL questionnaire presents adequate psychometric characteristics for use in patients with schizophrenia and bipolar disorder. The completion time of the questionnaire was low (<5 min) compared with longer questionnaires such as the UKU scale. Moderate-high correlations were found in both bipolar and schizophrenic patients between the TOOL and specific clinician-rated measures as the MADRS, PANSS, and YMRS, as well as a negative relationship with generic quality of life instruments (EQ-5 D and SF-6D), thus highlighting the convergent validity of this instrument. These correlations were also similar to those found in other studies analysing the association between generic and specific measures [31]. Criterion and discriminative validity were proven by the significant differences found in the TOOL between those patients with mild involvement and patients with moderate-severe symptoms. Thus, patients suffering a worse health status according to the CGI-S obtained higher scores in the TOOL, which indicates a more severe impact on their HRQoL, as has been previously underscored [32,33].

The one-dimensional construct of the TOOL, along with its internal consistency and test-retest reliability, have been proven, thus allowing clinicians to obtain a simple and global score (ranging from 8 to 32) with the same interpretation in terms of severity as the generalised UKU scale.

Despite these results, this study presents some limitations that should be considered. A ceiling effect was observed in two items: stiffness-tremor and dizziness-nausea. This may be due to the idiosyncrasy of the population sample collected in the present study: clinically stable patients under drug treatment, based mainly on atypical antipsychotic agents. Although similar ceiling effects have been reported in other studies [31,32], these items should be tested in further investigations with schizophrenic and bipolar patients showing a worse health status in order to test discriminative power. The characteristics of the sample may limit the scope of use of this measure. Therefore, it may be arguable whether patients with greater involvement (that is, patients with severe negative symptoms or patients suffering a maniac episode) could provide reliable reports when completing the questionnaire. Although there is evidence supporting the accuracy of such measurement, as has been commented above, the questionnaire should be applied within such populations to test its psychometrical properties. Secondly, it is necessary to analyse the sensitivity of the TOOL in detecting changes in longitudinal studies.

## Conclusions

The Spanish validation of the TOOL questionnaire shows appropriate feasibility, reliability, and discriminative performance as a patient-reported outcome to be used for the assessment of the impact of side effects on patient health-related quality of life. This information could be very important to improve therapeutic alliances and treatment adherence among patients with schizophrenia and bipolar disorder.

## Competing interests

ALM: Grants from Lilly, BMS-Otsuka, GSK, Sanofi, Astra Zeneca, Boehringer and Wyeth. Fees from Lilly, BMS-Otsuka, GSK, Sanofi, Astra Zeneca, Boehringer and Wyeth. Served on advisory boards for Lilly, GlaxoSmithKline, Servier and AstraZeneca. LC, TD, and JM are employees of AstraZeneca. The remaining authors have no conflicts of interest.

## Authors' contributions

ALM, JCL, JC, PR, LC, TD, and JM conceived the study design. JC and PR performed the statistical analyses. All authors made meaningful contributions to data interpretation. ALM, JC, and JM cowrote the final draft of the manuscript. All authors read and approved the final manuscript.

## Supplementary Material

Additional file 1 **TOOL Spanish version**. The Spanish version of the 'TOlerability and quality Of Life' (TOOL) questionnaire. Click here for file
