# Supplementary material for: Validation of a specific measure to assess health-related quality of life in patients with schizophrenia and bipolar disorder: the 'Tolerability and quality of life' (TOOL) questionnaire
Source: Ann Gen Psychiatry. 2011 Mar 11;10:6. doi: 10.1186/1744-859X-10-6 (PMC3062605; doi:10.1186/1744-859X-10-6)
Supplement: Additional file 1 — TOOL Spanish version. The Spanish version of the 'TOlerability and quality Of Life' (TOOL) questionnaire. [file 1744-859X-10-6-S1.PDF]

Marque con una cruz la casilla, de cada uno de los grupos de abajo, correspondiente a la afirmación que describa mejor su estado de salud a día de hoy.

Preocupación o desánimo

- ☐ No estoy preocupado o desanimado
- ☐ Estoy un poco preocupado o desanimado
- ☐ Estoy bastante preocupado o desanimado
- ☐ Estoy extremadamente preocupado o desanimado

Actividades cotidianas (trabajo, estudios, tareas del hogar, actividades familiares o de ocio)

- ☐ No tengo ninguna dificultad para realizar mis actividades cotidianas
- ☐ Tengo algunas dificultades para realizar mis actividades cotidianas
- ☐ Tengo bastantes dificultades para realizar mis actividades cotidianas
- ☐ Tengo muchas dificultades para realizar mis actividades cotidianas

Cansancio o debilidad

- ☐ No estoy cansado o débil
- ☐ Estoy un poco cansado o débil
- ☐ Estoy bastante cansado o débil
- ☐ Estoy extremadamente cansado o débil

Peso corporal

- ☐ No tengo sobrepeso
- ☐ Tengo un poco de sobrepeso

- ☐ Tengo bastante sobrepeso
- ☐ Tengo mucho sobrepeso

Rigidez o temblores

- ☐ No tengo ningún problema de rigidez o temblores
- ☐ Tengo algunos problemas de rigidez o temblores
- ☐ Tengo bastantes problemas de rigidez o temblores
- ☐ Tengo muchos problemas de rigidez o temblores

Inquietud o intranquilidad (dificultad para quedarse quieto)

- ☐ No estoy inquieto o intranquilo
- ☐ Estoy un poco inquieto o intranquilo
- ☐ Estoy bastante inquieto o intranquilo
- ☐ Estoy extremadamente inquieto o intranquilo

Función sexual

- ☐ No tengo problemas con el deseo ni con el funcionamiento sexual
- ☐ Tengo algunos problemas con el deseo o el funcionamiento sexual
- ☐ Tengo muchos problemas con el deseo o el funcionamiento sexual
- ☐ Tengo dificultades extremas para tener deseo o funcionamiento sexual

Mareos o náuseas

- ☐ No tengo mareos o náuseas
- ☐ Tengo algunos mareos o náuseas

- ☐ Tengo bastantes mareos o náuseas
- ☐ Tengo muchos mareos o náuseas

PUNTUACIÓN TOTAL:
